# A new strategy to prevent biofilm and clot formation in medical devices: The use of atmospheric non-thermal plasma assisted deposition of silver-based nanostructured coatings

**DOI:** 10.1371/journal.pone.0282059

**Published:** 2023-02-22

**Authors:** Tommaso Gallingani, Elisa Resca, Massimo Dominici, Giuliana Gavioli, Romolo Laurita, Anna Liguori, Giorgio Mari, Luca Ortolani, Eva Pericolini, Arianna Sala, Giulia Laghi, Tiziana Petrachi, Gaëlle Francoise Arnauld, Luca Accorsi, Rita Rizzoli, Vittorio Colombo, Matteo Gherardi, Elena Veronesi

**Affiliations:** 1 Department of Industrial Engineering (DIN), Alma Mater Studiorum-Università di Bologna, Bologna, Italy; 2 Technopole “Mario Veronesi” (TPM), Mirandola, MO, Italy; 3 Department of Medical and Surgical Sciences for Children & Adults, University-Hospital of Modena and Reggio Emilia, Modena, Italy; 4 B. Braun Avitum Italy S.p.A., Mirandola, MO, Italy; 5 Department of Chemistry, Alma Mater Studiorum-Università di Bologna, Bologna, Italy; 6 IMM-Consiglio Nazionale delle Ricerche, Bologna, Italy; 7 Department of Surgical, Medical, Dental and Morphological Sciences with interest in Transplant, Oncological and Regenerative Medicine, University of Modena and Reggio Emilia, Modena, Italy; 8 Advanced Mechanics and Materials, Interdepartmental Center for Industrial Research (AMMICIR), Alma Mater Studiorum-Università di Bologna, Bologna, Italy; Mohanlal Sukhadia University, INDIA

## Abstract

In industrialized countries, health care associated infections, the fourth leading cause of disease, are a major health issue. At least half of all cases of nosocomial infections are associated with medical devices. Antibacterial coatings arise as an important approach to restrict the nosocomial infection rate without side effects and the development of antibiotic resistance. Beside nosocomial infections, clot formation affects cardiovascular medical devices and central venous catheters implants. In order to reduce and prevent such infection, we develop a plasma-assisted process for the deposition of nanostructured functional coatings on flat substrates and mini catheters. Silver nanoparticles (Ag NPs) are synthesized exploiting in-flight plasma-droplet reactions and are embedded in an organic coating deposited through hexamethyldisiloxane (HMDSO) plasma assisted polymerization. Coating stability upon liquid immersion and ethylene oxide (EtO) sterilization is assessed through chemical and morphological analysis carried out by means of Fourier transform infrared spectroscopy (FTIR) and scanning electron microscopy (SEM). In the perspective of future clinical application, an *in vitro* analysis of anti-biofilm effect has been done. Moreover, we employed a murine model of catheter-associated infection which further highlighted the performance of Ag nanostructured films in counteract biofilm formation. The anti-clot performances coupled by haemo- and cytocompatibility assays have also been performed.

## Introduction

Nosocomial infections are primarily induced from bacterial colonization of medical device surfaces, to form a biofilm as a reservoir of pathogens often resistant to antibiotics therapy [1]. Lower respiratory and thorax infections, intra-abdominal infection and bloodstream infections (BSI) accounted for more than 70% of deaths attributable to antimicrobial resistance in 2019 over the world. The use of medical device such as the central venous catheters (CVC) represented the 40% of all primary BSIs [1].

Catheter-related bloodstream infections (CRBSI), one of the most severe infections in patients with haematological and oncological malignancies [2]. Represents up to almost half of BSI [2]. Besides the clinical impact, CRBSI are associated with prolonged hospitalizations, significant morbidity, increased health-care costs [3] and mortality [3].

Beside nosocomial infections, platelets activation and consequently thrombus formation is also reported increasing risk of atherosclerosis and thrombosis, as well as ischemia. Implantable and temporary medical devices such as cardiovascular medical devices and CVC are exposed to blood for weeks to years. Although these devices have improved the quality of or extended life for many patients, they cause thrombotic events necessitating the use of anticoagulant and/or antiplatelet therapies, which increase bleeding risks [4]. Research and development (R&D) has extensively been focused on material properties including surface chemistry, wettability, roughness, and charge [4] o mimic the endothelial layer reducing the platelet activation and ischemia risk but without successful results. Scientific community has been moved on surface coatings with anticoagulant, anti-bacteria properties [4] but the toxicity effect due to the release of the coating, as well as the dose needed to obtain the prolonged anti-bacteria effect, represented a challenge [5].

In this framework, developed from the pioneering works of Biederman [6] D’Agostino [7] and Yasuda [8] the surface polymerization carried out with low vacuum plasma and, more recently, with atmospheric pressure plasma has been exploited with the aim of improving the surface properties of biomaterials [9]. The plasma coating techniques is of great interest due to the mild conditions used to functionalize different thermo-sensitive materials. While low vacuum setups are widely explored and reported [10], researchers have recently improved atmospheric non-equilibrium plasma processes enabling the introduction or even support the *in-situ* nanostructured coating deposition [11–13]. Among the possible strategies, the use of precursors in aerosol form is receiving increasing interest [11]. In particular, Liguori et al. [14] reported on the possibility to use an ethanol aerosol flow as a carrier to enable the localized co-deposition of Ag NPs and polymeric coatings. Ag-containing coatings are by far the most prevailing antibacterial nanomaterials [15] due to the advantages of broad antibacterial spectrum efficacy, low systemic absorption of Ag+ ions with tissue cells, satisfactory stability [15]. However, Ag-containing coatings and released Ag+ may also affect the tissue cells, thus requiring the control and regulation of dosage to avoid cytotoxicity side effects.

In this study we carried out a wide investigation on silver-based atmospheric-pressure plasma deposited nanostructured coatings to gain insight on the anti-biofilm properties of Ag NPs, through plasma assisted reduction of a AgNO_3_ containing liquid aerosol. Synthesized particles were embedded in a multilayer coating deposited on 2D polyurethane substrate already in use for medical device manufacturing and selected as a gold standard; an organosilicon precursor was employed for the synthesis of the coating since this class of molecules is known for its suitability in atmospheric pressure plasma deposition due to enabling the production of thin films with an ample range of chemo-morphological characteristics as a function of process conditions [16,17]. Once completed the chemical and morphological analysis of deposited coatings and assessed their stability, biological assays were carried out to evaluate the substrates biocompatibility and anti-biofilm performances.

After the analysis of coating stability upon ethylene oxide (EtO) sterilization and soaking, we investigated the use of optimized process for mini catheter coating in order to processed biomaterial functionalities through a murine model of catheter-associated infection.

## Material and methods

### Plasma-assisted coating deposition on 2D substrate

#### Plasma source and process

**Fig 1:**
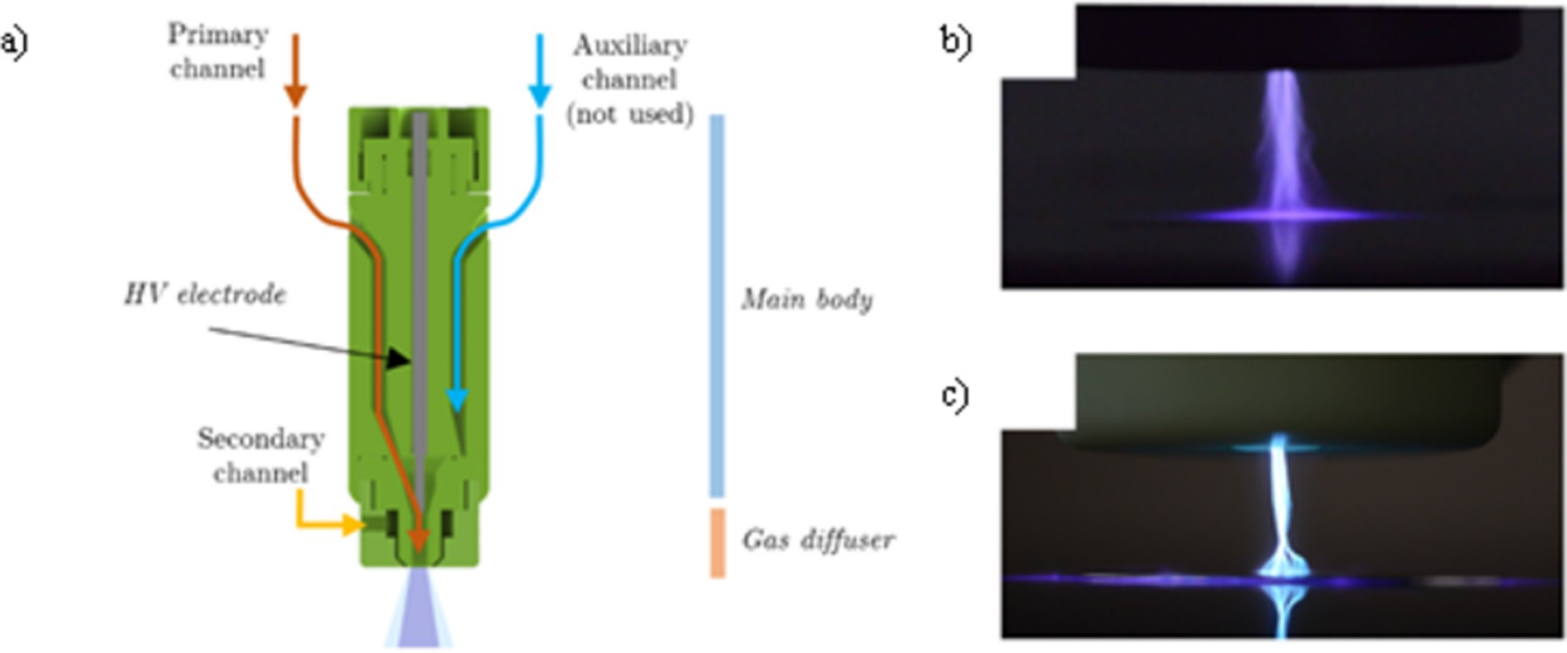
Plasma jet employed for the present study. In a) schematic; b) plasma discharge on dielectric substrate; c) plasma discharge on conductive substrate.

**Fig 2:**
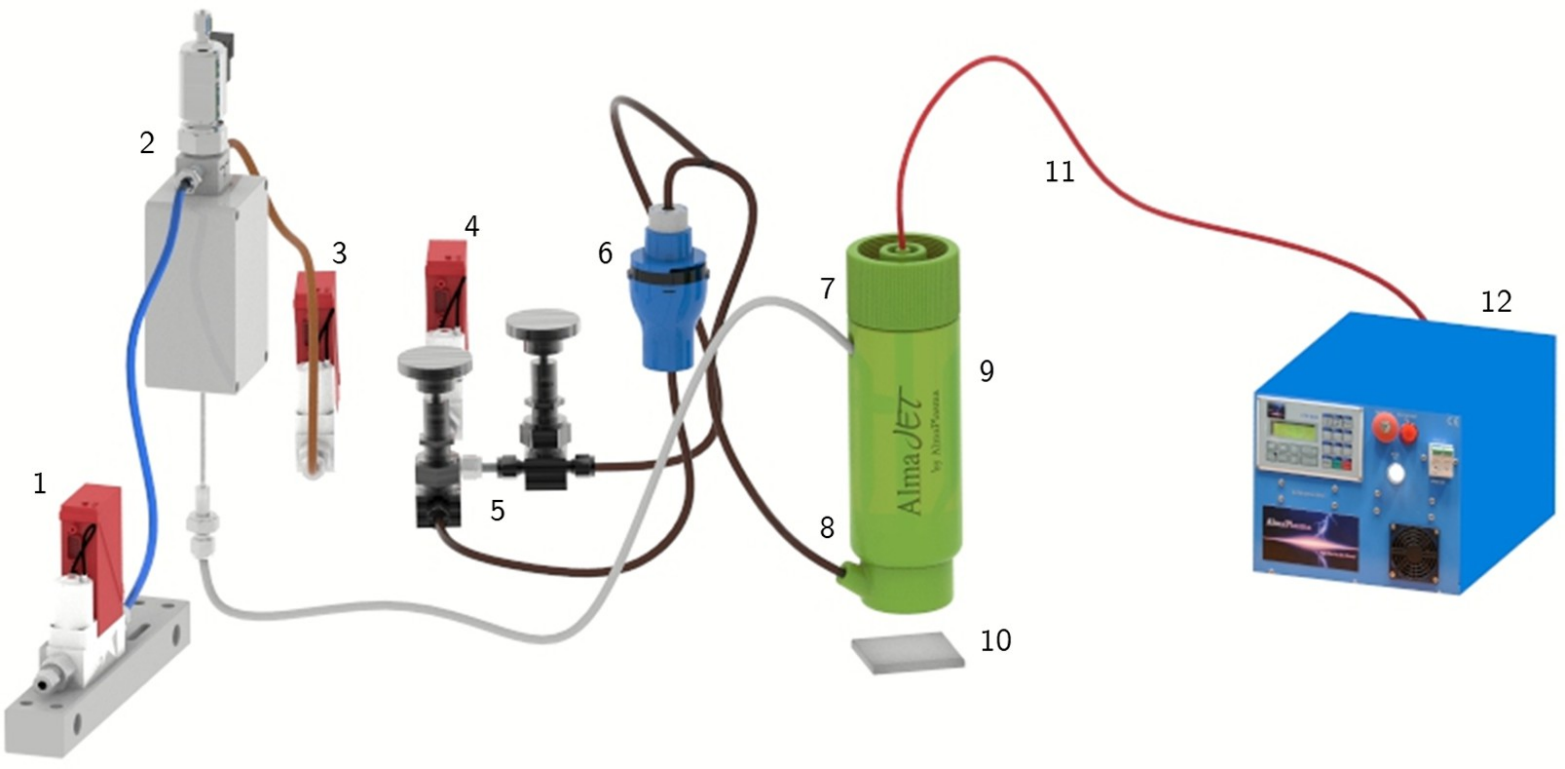
Schematic of setup used for the deposition of coating and synthesis of Ag NPs. 1) Liquid mass flow rate; 2) Controlled evaporation mixer; 3) Primary gas mass flow rate; 4) Secondary gas mass flow rate; 5) Bypass valves to support synthesis and deposition steps; 6) Nebulizer; 7) Primary gas connection; 8) Secondary gas connection; 9) Plasma source; 10) Sample; 11) High voltage cable; 12) High voltage pulse generator.

Coating deposition and in-flight synthesis of Ag NPs were carried out by means of a single electrode atmospheric pressure plasma jet (APPJ) developed at the Alma Mater Studiorum-University of Bologna, already studied and employed to support plasma assisted polymerization, nanostructured film deposition and liquid treatments (Fig 1) [14,18,19]. The plasma discharge was generated at the tip of the high voltage electrode where the primary argon (Ar) flow was introduced enabling the propagation of the plume through a small orifice. The plasma source was equipped with a specifically designed secondary gas diffuser suitable for the introduction of precursors directly in the plasma region. Gas flows were controlled by means of two mass flow controllers (El-Flow, Bronkhorst). The formation of a plasma plume at the exit of the plasma jet enabled the generation of a semi-controlled discharge, where the gas fluid dynamics was employed to limit ambient gas intake. A complete description of the gas fluid dynamics has been published by Colombo et al. [19,20]. The APPJ was driven by a commercial high voltage generator (AlmaPULSE, Almaplasma srl, Italy) working at 10 kV of peak voltage, with a pulse repetition frequency set at 14 kHz. Electrical characterizations of the plasma source were carried out measuring the voltage and current waveform on the high voltage cable by means of a high voltage probe (P6015A, Tektronix) and current probe (6585, Pearson). Real-time power measurements were performed processing the current and voltage waveforms acquired using an oscilloscope (DPO4043, Tektronix) connected to a computer through in-house developed MatLab^®^ script. Power delivered to the plasma source was calculated by integration of instantaneous power along the period taking into account a large number of acquisitions (~450). A schematic of the experimental setup used for coating deposition and particle synthesis is reported in Fig 2.

**Fig 3:**
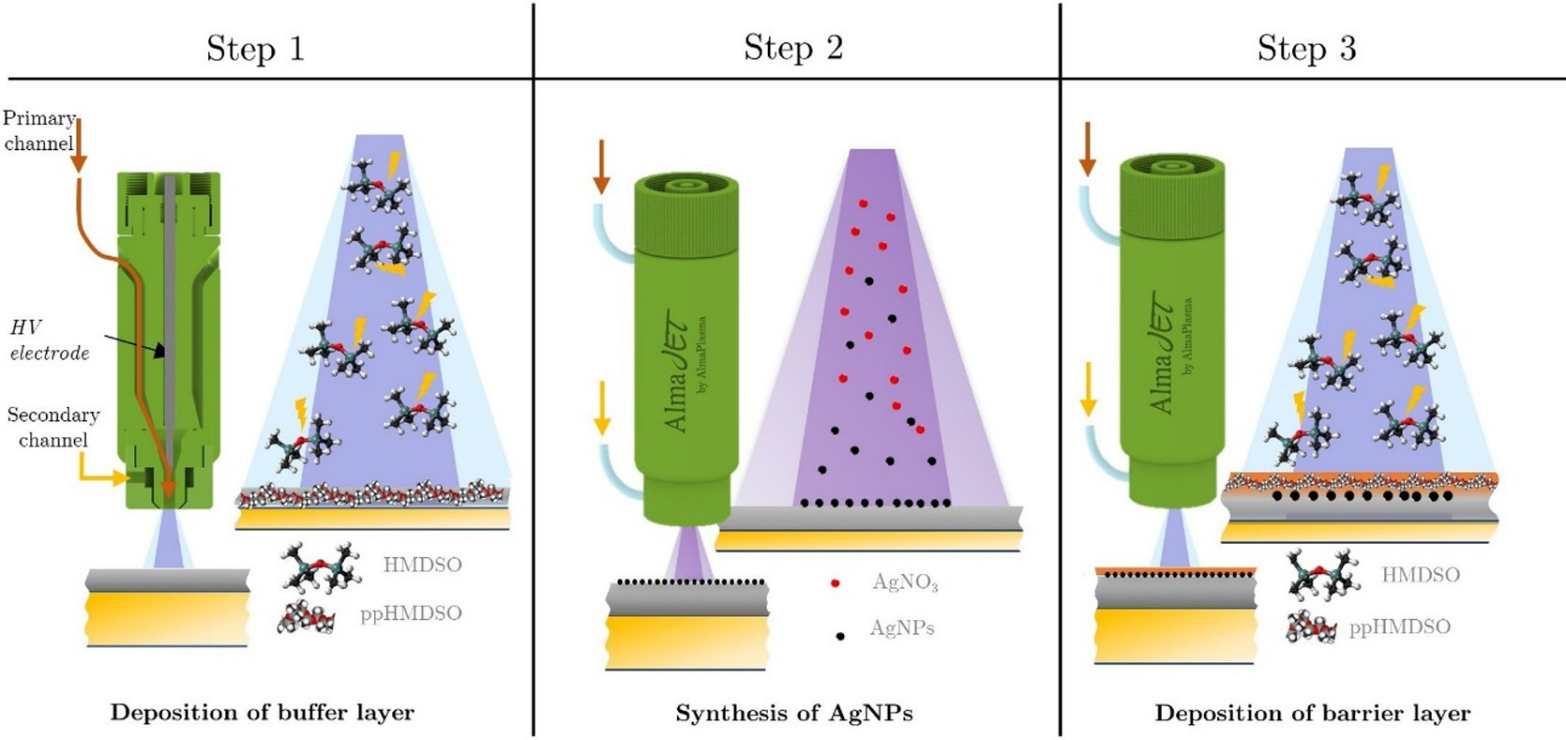
Schematic of plasma process for nanostructured coating deposition. In a) deposition of the buffer layer; b) in-flight synthesis and deposition of Ag NPs; c) deposition of the barrier layer.

Nanostructured coating deposition was carried out in three different process stages (Fig 3) that allowed the embedment of plasma synthesized Ag NPs between a buffer layer and an organosilicon barrier layer able to prevent particles release and dispersion in the liquid phase. A first buffer layer was deposited on polyurethane samples introducing 0.2 g/h of hexamethyldisiloxane (HMDSO, Sigma Aldrich) carried in the plasma discharge by 1.7 liter per minute (l/min) flow of Ar. An Ar flow of 2.4 l/min was introduced through the secondary gas diffuser, with the aim of increasing the discharge volume and, thus, the deposition area. The deposition of the buffer layer lasted 60 seconds. Afterwards, Ag NPs synthesis was performed injecting in the plasma region silver nitrate (AgNO_3_) containing droplets carried by 2.4 l/min flow of Ar: the aerosol was introduced in the discharge through the secondary diffuser. The concentration of the nebulized solution was kept constant at 250 mM for all the experiments: the solution was prepared dissolving AgNO_3_ in distilled water (Sigma Aldrich, HLPC grade) and stirring for 15 minutes at room temperature; the nebulization was performed trough a medical grade aerosol nebulizer (Aerea NEBJET, Moretti), with declared diameter of the produced aerosol particles in the range 2–3 um. Finally, the deposition of the barrier layer was carried out in the same operating condition used for the buffer layer deposition. The in-flight Ag NPs synthesis and barrier layer deposition lasted 300 and 30 seconds, respectively. A summary of all the operating conditions and parameters used in the experiments is reported in Table 1.

**Table 1:** Operating conditions.

	Buffer layer deposition	Ag NPs synthesis and deposition	Barrier layer deposition
*Primary gas flow*			
Ar flow rate	1.7 l/min	1.7 l/min	1.7 l/min
HMDSO flow rate	0.2 g/h	-	0.2 g/h
*Secondary gas flow*			
Ar flow rate	2.4 l/min	2.4 l/min	2.4 l/min
Precursor	-	250 mM AgNO_3_ aerosol	-
Treatment time	60 s	300 s	30 s
Plasma power	11.7 ±0.2W	9.2 ± 0.2 W	11.7 ±0.2W

In this table we report operating conditions used for plasma assisted coating deposition and Ag NPs synthesis.

Deposition of multilayer films was performed on polyurethane substrates widely employed in the biomedical field for the manufacturing of medical devices. It’s worth mentioning that usually central venous catheters (CVCs) are loaded with barium sulphate (BaSO_4_) NPs to ensure radiology detection of the medical device once placed inside the body. For this reason, coatings were deposited on polymeric plates (30x30x3mm^3^) produced by a molding process and loaded with BaSO_4_. Pristine substrates were placed 7 mm below the plasma source and exposed to the plasma discharge. Expanding from the APPJ orifice, the interaction of the plasma discharge and the treated substrate took place up to ~7 mm in the radial direction from the tip of the electrode (as can be observed in the pictures reported in the next paragraph and in literature [21], leading to a deposition area of ~150 mm^2^.

#### Chemical and morphological characterization

While chemical characteristics of the coatings were analyzed by means of Fourier transform infrared spectroscopy in ATR mode (ATR-FTIR, Agilent Cary 660), substrate morphological characteristics were investigated using a scanning electron microscopy and energy dispersive X-ray microanalysis (SEM-EDX, Phenom G2 ProX) on preliminary gold palladium sputter coated samples (SC7620 mini sputter coater, Quorum Technologies). Static contact angle (CA) measurements were performed by means of drop shape analyzer (DSA30, KRUSS): a distilled water droplet (2 μl) was deposited on the sample substrate and the contact angle was measured using the Young-Laplace method.

#### Water stability test

According to ISO-10993-13 [22] and ISO-3781 [23] the stability of deposited coatings was assessed through the immersion of the treated samples in 50 ml of phosphate-buffered saline solution (PBS) for 7 days in a shaking plate incubator at 37°C. After stability tests, coating morphological and chemical characteristic were investigated using ATR-FTIR, SEM and CA analysis. For each characterization technique, the analyses were carried out on two replicates.

#### In-vitro biological assay

With the aim of evaluating the hemocompatibility of the deposited coatings, dynamic blood contact tests were performed: plasma treated substrates were dipped in human blood and incubated at 37°C for 24 hours using an orbital shaker. Cell lysis of blood samples was evaluated by haemoglobin free assay (Hemoglobin Assay Kit Sigma MAK115) according to ISO10993-4 [24]. The value of free haemoglobin (free HGB) was then calculated through a calibration curve, as suggested by the assay procedure. After blood contact test, biomaterials were stained with hematoxylin/eosin: substrates were fixed in formalin (10% v/v, 15 minutes), stained with hematoxylin, washed with tap water, counterstained with eosin (20 seconds) and finally washed in double distilled water (ddH2O).

Anti-biofilm performances were evaluated after incubation of the treated substrates in a four strains broth bacterial culture (*Bacillus subtilis*, *Staphylococcus aureus*, *Pseudomonas aeruginosa*, *Enterococcus hirae* 1:1:1:1, 10^8^ CFU/ml) for 10 days at 37°C, according to the methodology proposed by Petrachi et al. [25] Crystal violet (CV) staining was used to estimate biofilm formation on the coated biomaterials: substrates were fixed in methanol (-20°C) for 2 minutes, washed in ddH_2_O and dipped in crystal violet (0.4%) for 5 minutes.

After substrate staining procedures, clots and biofilm formation was evaluated by means of stereo microscope imaging (Microscope for Large Fields AxioZoom V16, Carl Zeiss Microscopy GmbH). All the analyses were carried out on three biological replicates. Staining controls for untreated substrates are available in the Supporting Information section (S1 Fig).

### Plasma assisted coating deposition on mini catheters

#### Plasma process

**Fig 4:**
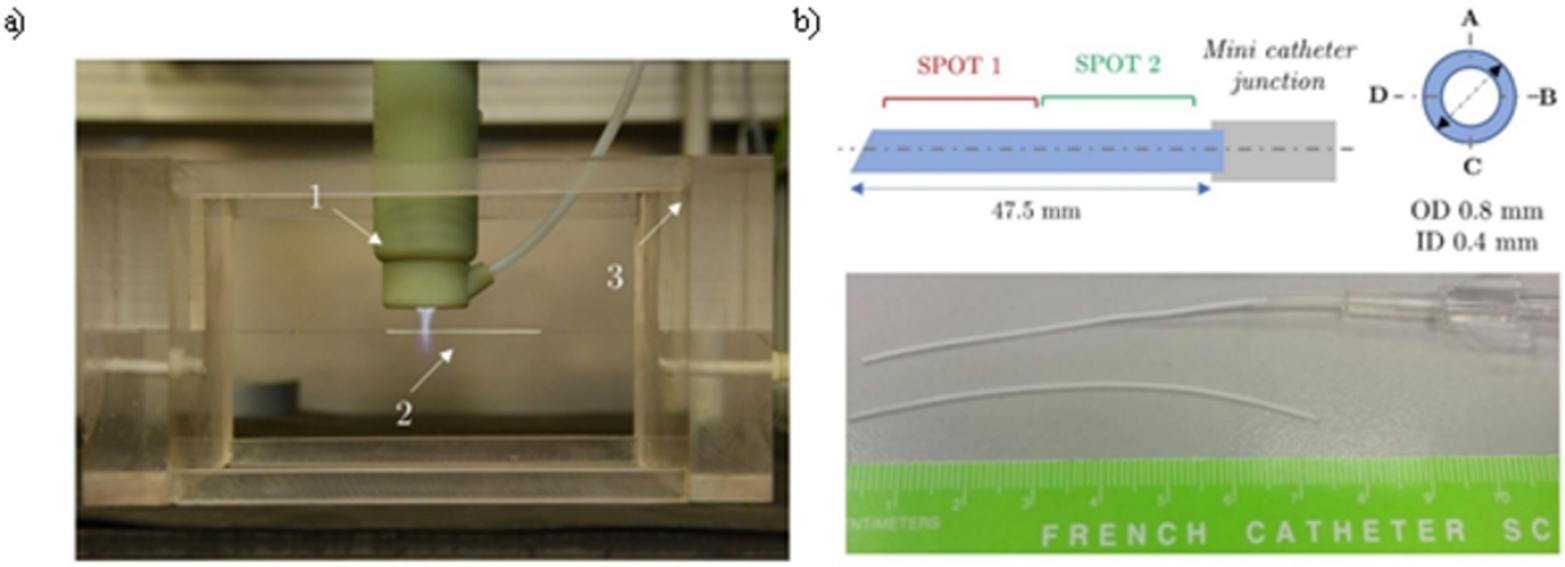
Plasma process for the deposition of nanostructured coating on mini catheters. In a) coating deposition on mini catheter (1 Corona Jet, 2 Mini catheter, 3 Sample holder); b) schematic and picture of the used mini catheter.

Following the promising results achieved in terms of anti-biofilm and anti-clot properties with respect to the 2D substrates, polyurethane mini catheters (50 mm length, OD 0.8 mm, ID 0.4 mm) were coated using the plasma assisted process previously described. The sample was treated by using a specifically designed holder, able to guarantee the processing of the entire catheter surface. Process was carried out following the three steps reported below:

1. Step 1: Deposition of buffer layer coating (60 s) for each side of the catheter, rotating the substrate by 90° every 60 s (total treatment time 4 x 60 s);
2. Step 2: Synthesis and deposition of Ag NPs (120 s) for each side of the catheter, rotating the substrate by 90° every 120 s (total treatment time 4 x 120 s);
3. Step 3: Deposition of barrier layer coating (30 s) for each side of the catheter, rotating the substrate by 90° every 60 s (total treatment time 4 x 30 s);

The operating condition for the three different steps has been already reported above (Table 1). The deposition of multilayer coatings (buffer layer deposition, Ag NPs synthesis, barrier layer deposition) was performed in two different spots along the length of the mini catheter, located respectively at 10 mm and 30 mm from its tip (Fig 4).

The distance between the plasma source and the catheter was kept constant at 7 mm. The treatment time was set to 60 seconds and 30 seconds respectively for buffer layer and barrier layer deposition. Ag NPs were in-flight synthesized introducing 250 mM of AgNO_3_ aerosol containing droplets inside the plasma discharge for 120 s.

#### Chemical and morphological characterization

After deposition, the treated catheters were sterilized using ethylene oxide (EtO): the sterilization process lasted for 20 hours and was performed at 44°C in a 10% ethylene oxide and 90% CO_2_ atmosphere. Coating stability upon sterilization and immersion in tryptic soy broth (TSB) solution (72 hours at 37°C) was assessed by means of SEM analysis. Furthermore, microanalysis was employed to qualitatively confirm the presence of embedded Ag NPs after sterilization and stability test.

#### *In-vitro* biocompatibility assay

Finally, in-vitro compatibility of treated mini catheters was assessed by MTT test according ISO 10993–5 [26]: after biomaterial elution in cell culture medium for 24 hours at 37°C, the eluate was added to L929 culture and cell viability was assessed by colorimetric evaluation through MTT reduction (Microplate Reader, Perkin Elmer). As suggested by the standard, latex and high-density polyethylene (HDPE) were used as positive and negative controls, respectively. All samples were reported as % of viability.

#### Murine model of catheter-associated infection

Anti-biofilm performance of treated mini catheters were evaluated by means of in-vivo mouse model of catheter-associated infection using the experimental approach reported by Kucharíková et al. [27]. The bioluminescent bacterial strain *Pseudomonas aeruginosa* (P1242) (BLI-Pseudomonas) [28] was employed to contaminate the mini catheters implanted in mice. This strain was engineered to express both the luciferase gene and substrate under the control of a constitutive P1 integron promoter, in order to constitutively produce a detectable bioluminescent signal. Briefly, sterilized mini catheters (length 0.5 mm) were coated with fetal bovine serum (FSB), soaking the catheters overnight at 37°C in static conditions. Then, BLI-*Pseudomonas* overnight cultures (5 x 10^4^ cells/ml, in TSB plus 2% sucrose) were added and an additional 90 min incubation was performed. Then, mini catheters were washed twice with PBS at RT and implanted subcutaneously in female fvb/n mice (8 weeks age), as described elsewhere with minor modifications [27]. Mice were acclimatized for a week before starting the experiments. Mice were kept in individually ventilated filter top cages with free access to standard food and water *ad libitum*. All efforts were made to minimize suffering during experiments.

For each animal model, three contaminated catheters were introduced in a subcutaneous tunnel created on the back of immunosuppressed mice by surgery under anesthesia (i.p. injection of Tiletamine/Zolazepam-Xylazine, 50–5 mg/kg). Two- and six-days post-implant, mice were anesthetized, as above described, and imaged using IVIS camera system (Xenogen). Total photons flux emission from the Region Of Interest (ROI) was quantified using Living Image software package (Xenogen) and expressed as Relative Luminescence Units (RLU). After the final imaging time-point, the mice were humanely sacrificed by Quietek 2 CO_2_ (carbon dioxide) camera (Quietek™ Next Advance, Inc.). The Quietek 2 camera can deliver carbon dioxide to mice in accurate manner maintaining ethical standards. All efforts were made to minimize suffering during experiments.

**All the procedures involving animals and their care were conducted in conformity with national and international laws and policies and were approved by Italian Ministry of Health through the Ethical Committee (1017/2015_Pr 23/9/2015).** The animals were housed in the animal facility of the University of Modena and Reggio Emilia (Centro Servizi Stabulario Interdipartimentale, BIOSTAB, Authorization number 268/2011-A).

#### Statistical analysis

For *in vivo* experiments, RLU data from each experimental group were tested for normal distribution by Shapiro-Wilk test. Statistical analysis was performed using two-tailed Student’s T test by using GraphPad prism 8. Values of *p<0.05 were considered significant.

## Results and discussion

### Plasma assisted coating deposition on 2D substrate

**Fig 5:**
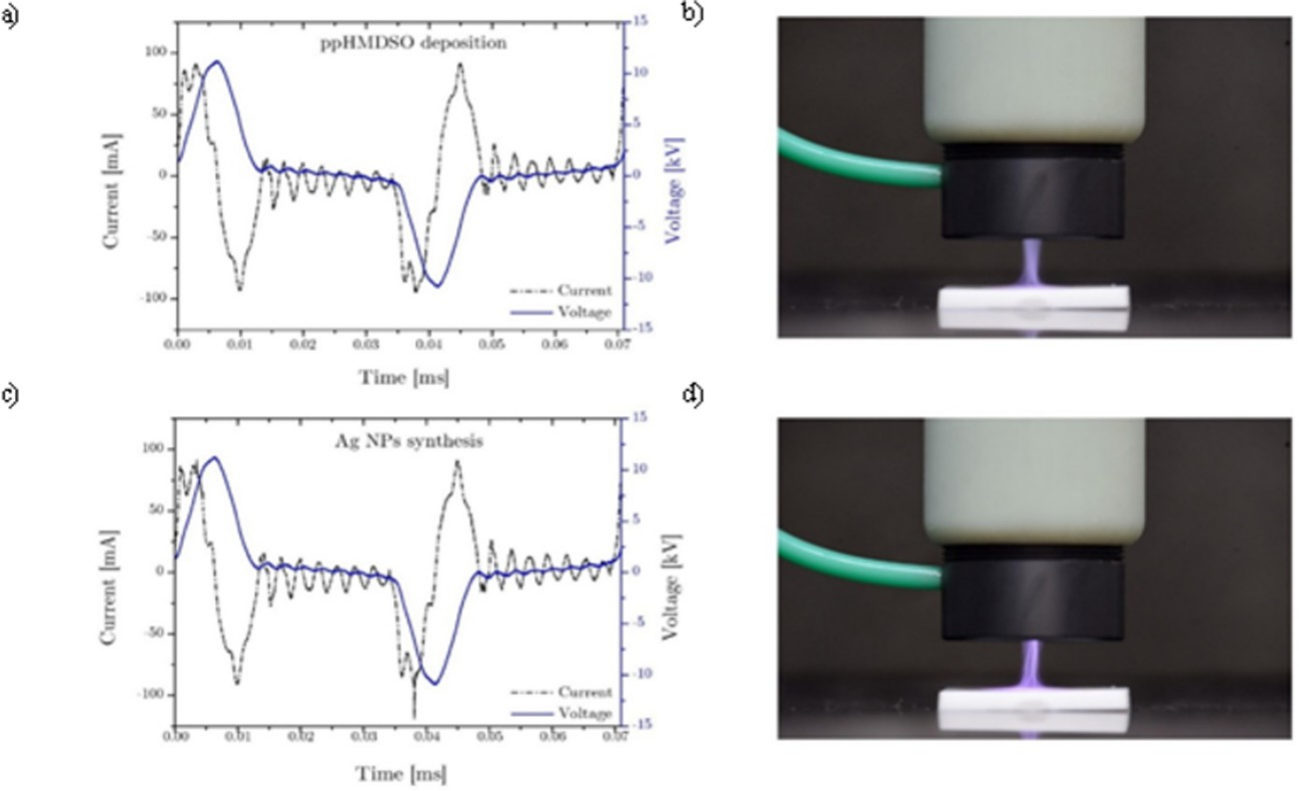
Voltage-current waveforms and picture of the plasma discharges. In (a,b) ppHMDSO deposition; in (c,d) Ag NPs synthesis.

Electrical power delivered to the APPJ was measured during all the steps of the multilayer coating deposition. Recorded data, reported in Table 1, outlined that the coating deposition took place at higher power value (11.7 W) with respect to the Ag synthesis step (9.2 W). The slight decrease of plasma power for NPs synthesis phase can be ascribed to the presence of water vapor that increased the breakdown voltage, thus lowering the energy that can be transferred to the gas phase. Even if the voltage-current waveforms, reported in Fig 5A and 5C, were characterized by similar trends, it is important to highlight that the discharge appearances for the two phases are different: while a glow-like discharge was achieved when the HMDSO precursor was introduced in the plume (Fig 5B), the Ag NPs synthesis process was characterized by a filamentary discharge where streamers propagated from the electrode tip to the substrate (Fig 5D). As reported in literature [29] the presence of vapor molecules during the deposition process can be accounted for the decrease of the electron temperature and the increase of the energy transferred to heavier species through collisions, enabling the transition to glow-like discharges [30,31]. Moreover, as reported by Tardiveau at al. [32] the introduction of droplets in the plasma discharge can locally affect the electric field, allowing the formation of streamers at lower voltage and affecting the discharge propagation.

**Fig 6:**
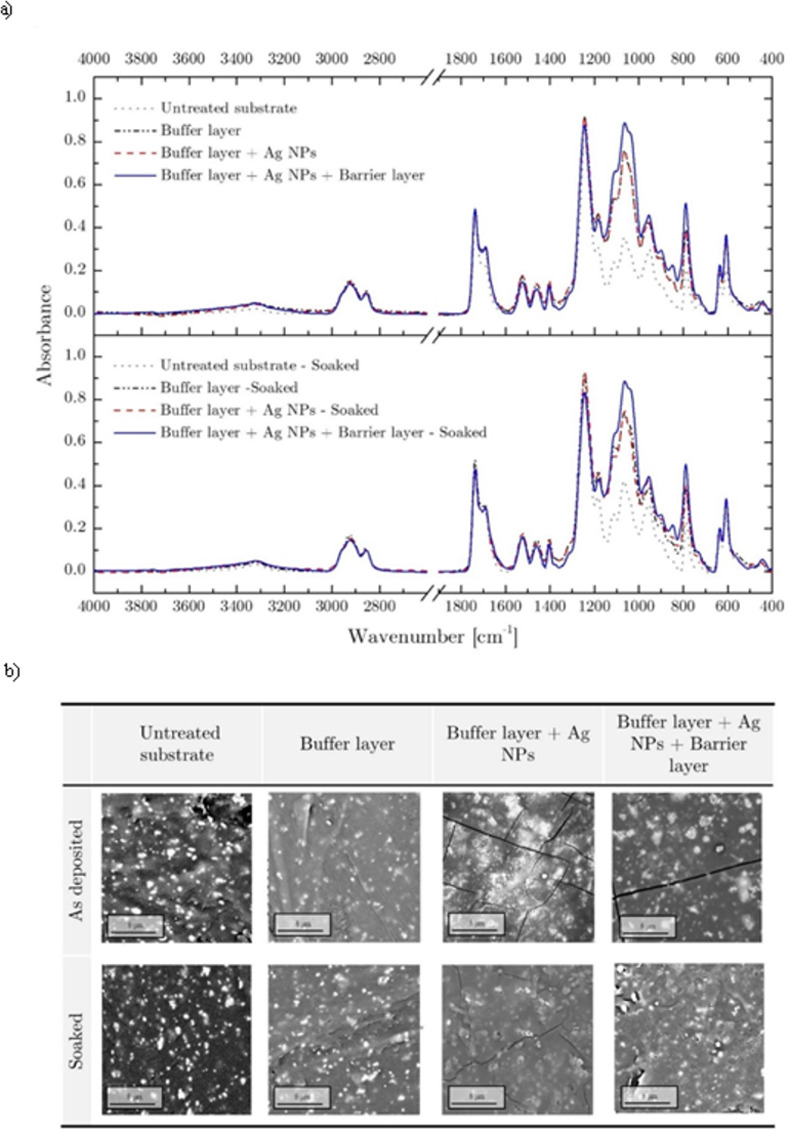
ATR-FTIR (a) and SEM (b) analysis. Representative analysis of deposited coatings before and after stability test.

In order to investigate the chemical and morphological properties of deposited coatings, ATR-FTIR analyses were performed after each deposition/synthesis step (Fig 6). The analysis of pristine sample spectrum highlighted the presence of CH_2_ (2864 cm^-1^), C-O (1713 cm^-1^) and N-H/C-N (1241 cm^-1^) bands, thus confirming the polyurethane structure of the material. With respect to that, the treated samples on which a buffer layer was deposited exhibited a characteristic peak centered at 1020 cm^-1^ that can be ascribed to Si-O-Si stretching vibrational mode [33]. The lower absorption bands at 445 cm^-1^ and 790 cm^-1^ were related to Si-O-C asymmetric and Si-CH_3_ rocking vibration [34]. While the deposition of Ag NPs did not affect the IR spectrum of the buffer layer, the deposition of the barrier layer led to higher absorbance value; in line with the presence of a thicker coating [35]. Although the presence of characteristics organo-silicon peaks confirmed the deposition of plasma polymerized HMDSO with a retention of C bonds [36,37], the IR spectrum of untreated material partially covers the organic characteristic peaks usually used to fully address organic-inorganic properties of the coating [37]. For this reason, water CA measurements were carried out to gain insights into the chemical composition of the coating. The possibility to infer the organic or inorganic nature of plasma polymerized coatings from WCA measurements for the case of HMDSO as precursor was previously demonstrated by Morent et al. [33]; the authors reported on the different wettability of coatings deposited through plasma polymerization of HMDSO in an oxygen-free environment (CA~100°) with respect to the ones processed when oxygen/air was added to the discharge (CA~20°). The presence of oxygen molecules indeed increases the fragmentation of the monomer and enables the formation of higher number of O-Si-O bonds, leading to inorganic and wettable films. Our data, reported in Table 2, highlighted that the deposited coatings were characterized by higher contact angle (lower surface energy) with respect to the pristine substrate. Even if not statistically significative, the increase of contact angle of the sample where Ag NPs were deposited on the buffer layer can be ascribed to changes in surface morphology, as reported by Yuce at al. [38] and accordingly to Wenzel [39]. Finally, the deposition of the barrier layer further decreased the surface energy leading to a CA of 101°. The hydrophobic behavior of the deposited coating, together with the presence of CH_3_ bonds highlighted in IR spectra, supported the hypothesis of the deposition of organic coating, characterized by a good retention of CH functionalities. Achieved results are in line with what was reported by Morent et al. [33] and, since our process was carried out in “semi-controlled” environment where the presence of ambient air (and therefore oxygen) was limited thanks to gas fluid dynamics, the high CA obtained for coated substrates confirmed the organic PDMS-like nature of deposited coatings.

**Table 2:** Water contact angle (WCA).

	WCA
*Untreated substrate*	75.1±2.3°
*Buffer layer*	95.5±1.9°
*Buffer layer+AgNPs*	99.2±3.1°
*Buffer layer+AgNPs+Buffer layer*	101.4±0.7°
*Buffer layer+barrier layer*	98.8±3.1°

**Fig 7:**
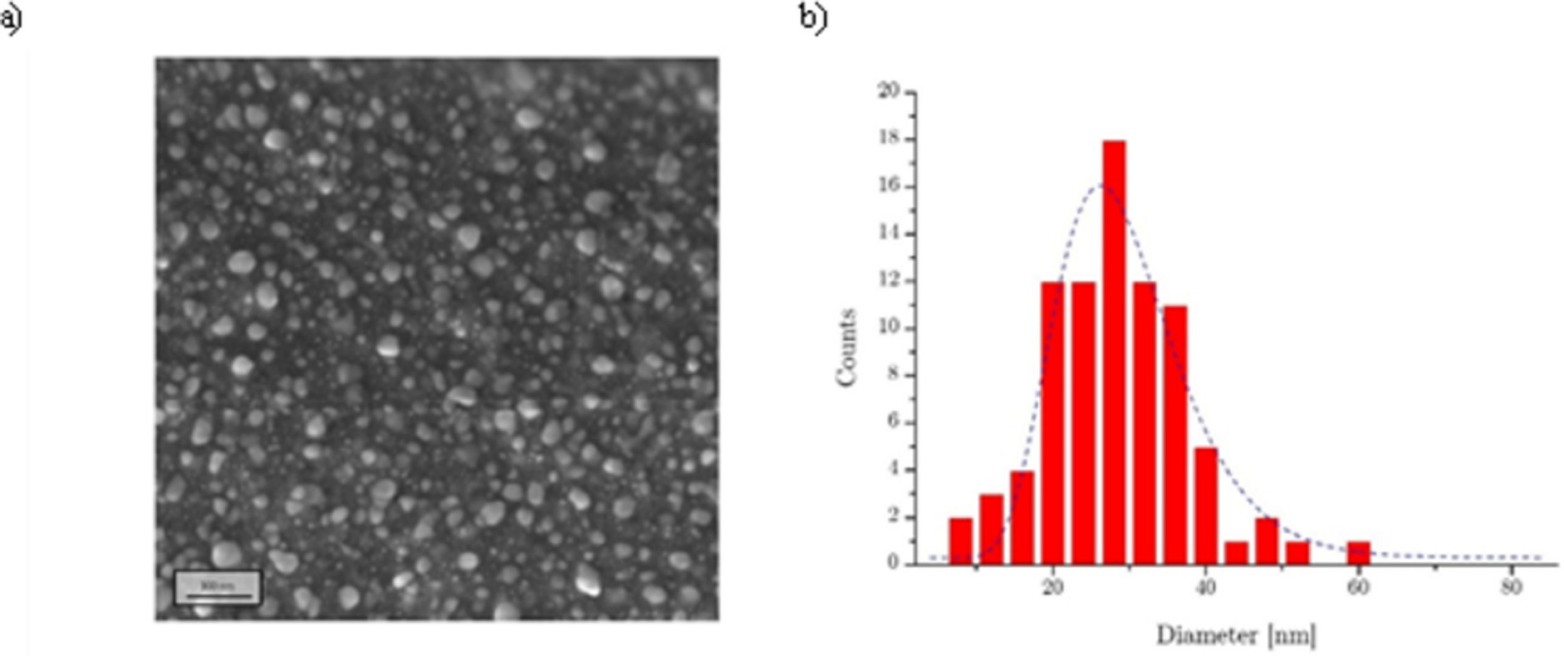
FE-SEM analysis. In (a) an image of Ag NPs deposited on glass slide and in (b) the synthesized particle distribution.

Chemical and morphological analysis were completed investigating the surface characteristics of deposited coatings using SEM and microanalysis techniques. As reported in Fig 6B, the presence of defects resulting from the molding process increased the surface roughness of the pristine sample. Moreover, the picture outlined the presence of BaSO_4_ particles, confirmed by EDX microanalysis (S2 Fig). When the buffer layer was deposited on the untreated samples, the sample exhibited a smoother surface, consistent with polymeric film deposition. The presence of Ag NPs after the synthesis step was confirmed both from SEM and EDX analysis. In particular, Ag NPs deposited on the buffer layer were widely spread on all the treated samples and were characterized by nanometric dimension. Nanostructure dimensions were analyzed by means of a high-resolution SEM-FEG, able to reach higher magnification and better resolution with respect to the desktop SEM used for coating analysis. Microscopy analyses were carried out on Ag NPs deposited on glass slides (with the aim of removing pristine substrate background morphology) and highlighted the synthesis of NPs with a broad particle sized centered at 28.4 nm (Fig 7). Moreover, even if not fully detectable because of limited instrument resolution, the presence of small particle (<10 nm) was found. The morphology of particles synthesized and deposited on the sample was in line with what was reported by Hong et al. [40]. Droplets to particles conversion in plasma flow-through reactor is still largely unexplored and many studies are currently investigating this synthesis process [41].

**Fig 8:**
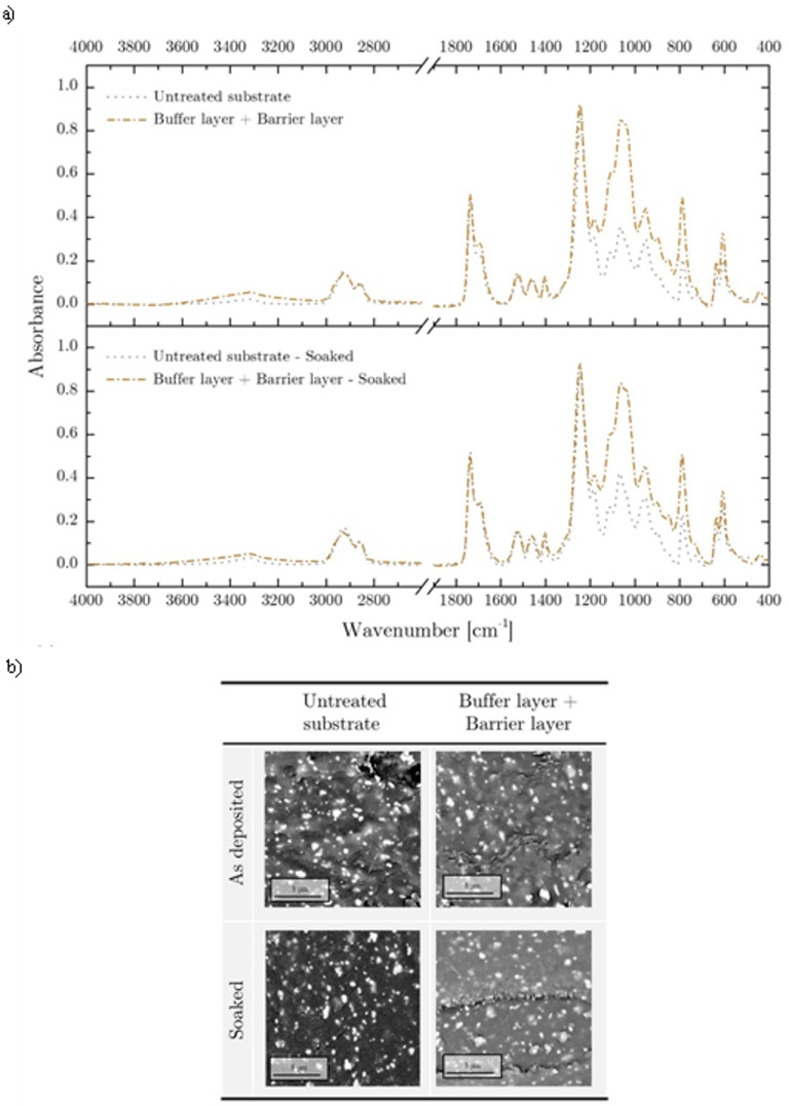
ATR-FTIR (a) and SEM (b) analysis. ATR-FTIR and SEM analysis of buffer layer + barrier layer sample before and after stability test.

To prevent nanoparticles dispersion in the biological environment, a polymeric barrier layer was deposited on Ag NPs. The SEM analysis outlined that the coating embedded the deposited particles in the ppHMDSO matrix. A deeper morphological analysis of the coating after Ag NPs synthesis and barrier layer deposition revealed the presence of cracks. With the aim of providing insight on cracks formation these results were compared with a control experiment where only buffer and barrier layer where deposited. Results (buffer layer + barrier layer, Fig 8) outlined the lack of cracks where only polymeric coating were deposited, suggesting that the presence of cracks was mainly due to the synthesis phase. A possible explanation of the cracks formation can be ascribed to the higher discharge temperature when water droplets (and water vapor) were introduced in the discharge [42] indeed thermal stress of the deposited coatings and treated substrates could have induced crack and defect production [43]. Even if defects of plasma coating deposition are usually undesired [44,45] Nikiforov et al [46,47] reported on the key role of cracks for the delivery of silver ions from nanostructured films deposited on non-woven fabrics.

In the perspective of a clinical application, beside antibacterial properties, deposited film should exhibit stability towards delamination, that is known to be responsible for medical devices failure and medical complications. For this reason, according to regulations [22,23] we investigated coating stability upon liquid solution immersion. IR spectra (Fig 6A) collected from treated samples after 7 days of stability test at 37°C outlined a limited reduction of coating characteristic peaks with respect to each *as deposited* film. While the presence of Si-O-Si, Si-O-C Si-CH_3_ highlighted the stability of plasma polymerized coatings, the small reduction in peak absorbance can be related to morphological surface modification due to liquid permeation, generally described as swelling. This hypothesis was supported by the result of SEM analyses which outlined changes in coating morphology (Fig 6B). After the prolonged soaking in the liquid solution, the buffer and barrier layers displayed a slight variation of the superficial roughness, leading to smooth and swollen surface. The key role of the barrier layer can be highlighted comparing the SEM images of Ag NPs deposited coatings with and without the upper protective layer. The presence of the barrier layer prevents the dispersion of Ag NPs in the liquid environment, maintaining a high level of Ag retain after immersion while controlling the Ag ions release, as already discussed in the literature [46,48,49].

Following the assessment of coating stability, the biological performances of plasma treated sample were assessed by means of dynamic blood contact test and biofilm adhesion assay. In this framework, we tested the properties of three different types of substrates: anticlot and antibiofilm performances were analyzed on untreated samples, polymeric (buffer layer + barrier layer) and nanostructured (buffer layer + Ag NPs + barrier layer) coatings. In this way, we separately investigated the role of Ag NPs with respect to the polymeric matrix. It’s also worth remembering that the untreated substrate is already in use for the production of medical devices (in particular of central venous catheters) and can be thereafter used as a reference gold standard.

**Fig 9:**
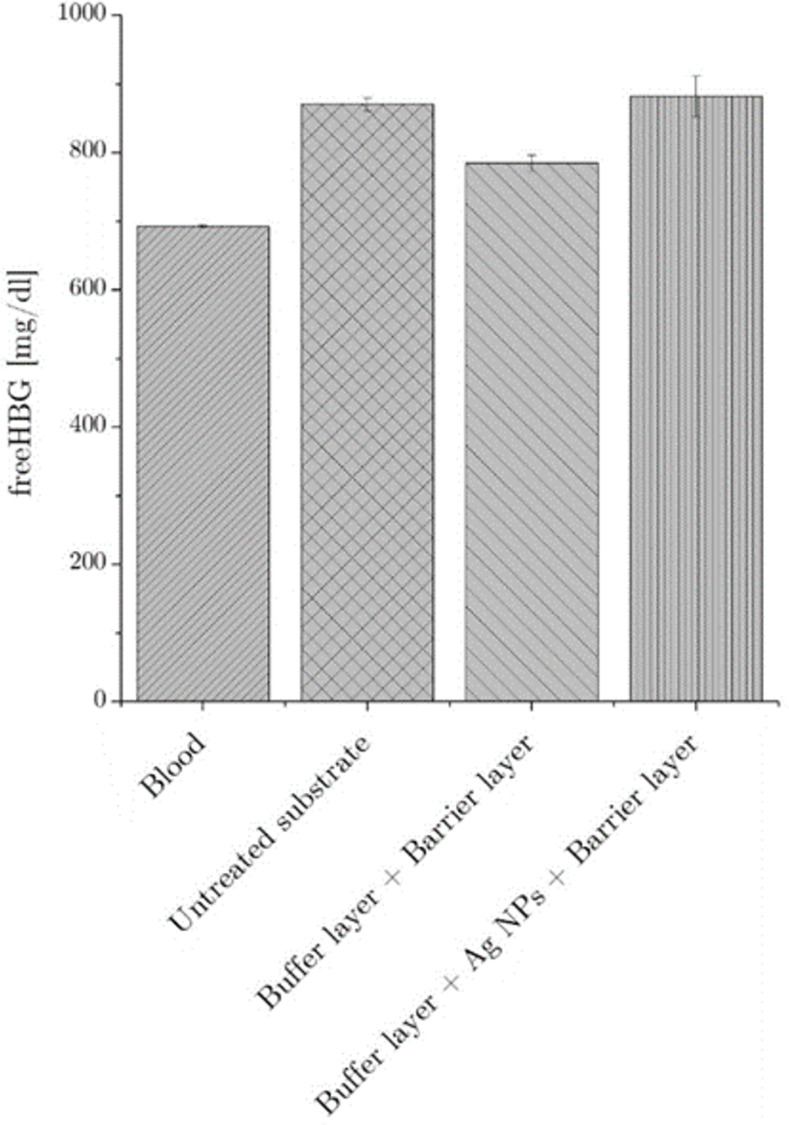
Evaluation of free haemoglobin. The graph represents free haemoglobin content after blood-biomaterial dynamic contact test. No significative differences were found between blood and treated materials.

The dynamic blood contact test was used to investigate both the hemocompatibility and the surface clot formation of deposited coatings, in the perspective of future *in-vivo* assay and application. While blood coagulation induced by shear forces between biomaterials surface and liquid phase should be kept under control to prevent thrombosis [50], clots formation has been reported as one of the major causes of device failure and biofilm proliferation [51]. For this reason, after 24 hours of contact with treated biomaterials, the free hemoglobin (freeHBG) content of blood samples was assessed using a colorimetric technique. It is worth remembering a higher content of freeHGB can be related to red cell lysis and to a higher coagulation rate. As reported is Fig 9, the deposition of the only polymeric coating (buffer layer+barrier layer) reduced the cell lysis and therefore increased the hemocompatibility of the treated samples. Conversely, the introduction of the Ag nanoparticles in deposited coatings (buffer layer + Ag NPs + barrier layer) displayed the same level of blood hemolysis of untreated substrate, our gold standard. Generally, obtained results showed that blood contact with treated and untreated substrates increase the freeHGB content. A possible explanation of the different hemolysis rate can be carried out, following what has been already reported in literature; according to what was discussed by Offeman [52], the dynamic contact between biomaterial and blood increases red cells stress and induces the hemolysis. More recently, other studies [53,54] highlighted that, beside shear forces, also surface roughness has an important effect on hemolysis rate. These findings supported the achieved experimental results and can also explain the decrease of free HBG when the polymeric coating was applied to the pristine substrate: the decrease in surface roughness, as discussed previously, can be accounted for the reduction of the shear-induced cell stress, limiting the hemolysis rate. Even if polar opposite views have also been reported regarding the effect of Ag NPs on blood hemolysis [55–57] recent works [58,59] have clarified that the release of silver ions induced direct platelets and red cells stress. The slight increase of hemolysis rate measured for the nanostructured coating corroborates these findings. We can therefore highlight that the deposited coating displayed an adequate level of hemocompatibility keeping under control the material-induced stress, even when the Ag NPs were embedded in the film matrix.

**Fig 10:**
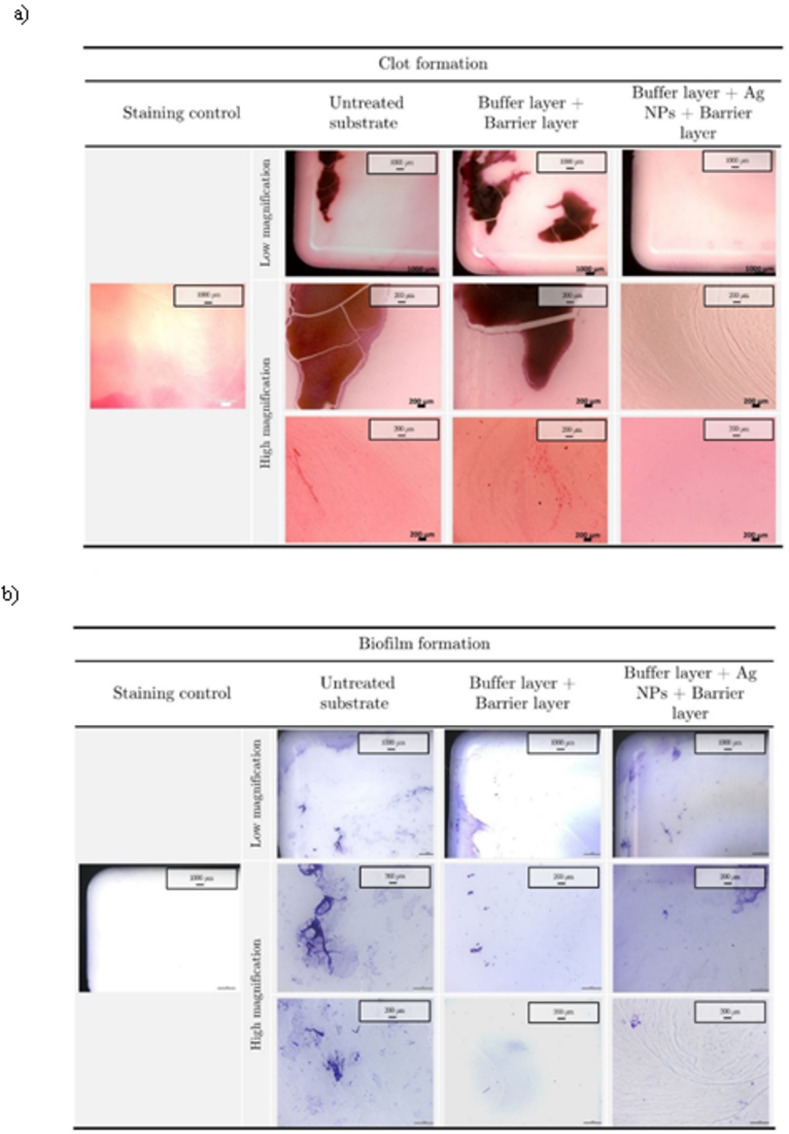
Hematoxylin/Eosin and crystal violet staining. Images of untreated and treated biomaterials stained with hematoxylin/eosin and crystal violet after blood (a) and bacterial broth incubation tests (b), respectively. The first line (low magnification) represents the edge of the biomaterial, while the high magnification is about the edge and the center of the biomaterial respectively.

The analysis of blood-biomaterial contact test was completed staining the substrate surface in order to evaluate the formation of clots. According to low magnification images of the stained samples, reported in Fig 10A, nanostructured coatings were able to reduce the formation of clots with respect to the pristine and polymeric coated substrates. The analysis of higher magnification fields of view highlighted that when Ag NPs were embedded in the coating matrix, the adhesion of procoagulant fibrine/platelets and the presence of small clots was completely avoided. A comparison with results on hemolysis rate underlined that the higher cell lysis for nanostructured coatings corresponded also to lower clots formation. Although the complex pathways of clot formation on biomaterials surface is a function of surface-blood interaction and it’s not the focus of this study, our results were in line to what was presented by Stevens et al. [59]. According to our findings, the authors reported on the increase of hemolysis rate and the contextual reduction of platelets adhesion when nanostructured Ag coated catheters were put in contact with human blood. Even if the mechanism was not fully described, the decrease of platelets adhesion can be accounted as the main reason for a reduction in clot formation on silver coated samples [47].

Following the assess of hemocompatibility, biofilm formation on biomaterials was investigated after 10 days of incubation in bacterial broth culture at 37°C. The use of crystal violet staining enabled the identification of bacterial colonies and the biofilm matrix. Stereo microscope imaging (Fig 10B) highlighted the presence of bacterial cells colonizing the surface of the untreated substrate. Conversely, the deposition of a polymeric coating strongly reduced bacterial colonization, hindering the formation of biofilm. The introduction of Ag NPs in the deposited film further improved the anti-biofilm performances, reducing the microbial adhesion, particularly in the central region of the sample where nanoparticles were deposited. The significative reduction of bacterial adhesion on the treated samples can be ascribed again to the combined role of surface properties and Ag toxicity. The deposition of polymeric coatings indeed decreased the surface roughness and led to a modification of the surface free energy/charge, which are among the causes of microbial adhesion and biofilm formation on medical devices [51]. Furthermore, the antibacterial properties of Ag NPs [60] embedded in the nanostructured coating contributed to interfere with bacterial viability, improving the performances of the coating [61].

The results discussed above underline that deposited nanostructured coatings were characterized by anti-clot and anti-biofilm performances. While the use of a thin barrier layer prevented Ag NPs dispersion during blood contact test, keeping under control the hemolysis rate, the release of Ag ions through the polymeric matrix hindered bacterial growth, adhesion and biofilm formation.

### Plasma-assisted coating deposition on mini catheters

**Fig 11:**
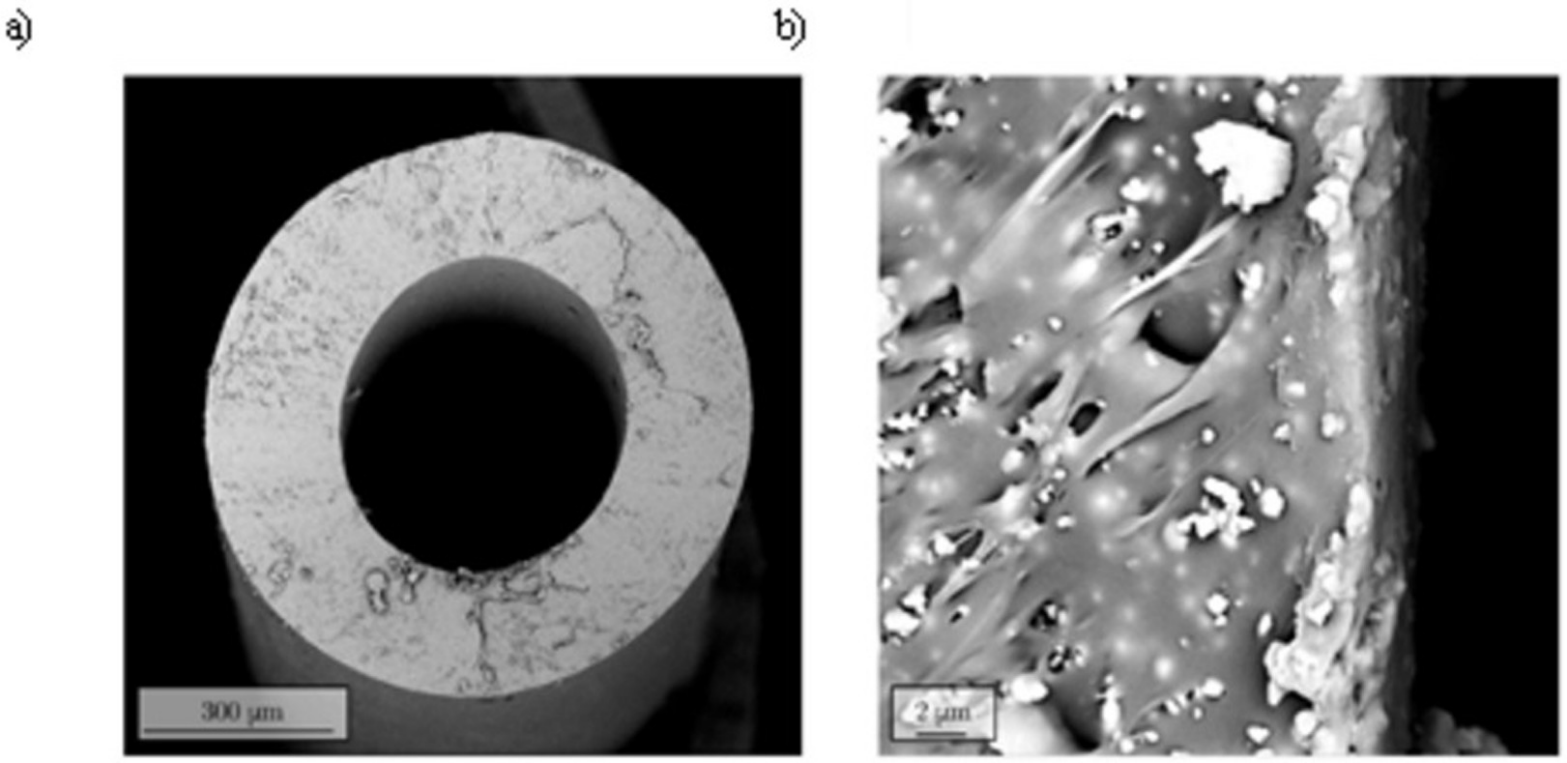
SEM images. Representative SEM images (a, b) of mini catheter side view after coating deposition.

Following the promising results obtained on bidimensional substrates, a new experimental setup was developed with the aim of coating the surface of mini catheters for *in-vivo* testing. The chemical and morphological analysis of treated samples was carried out using SEM-EDX all along the mini catheter length. SEM images (Fig 11) highlighted the presence of a coating in all the analyzed fields of view. The nanostructured nature of the film was confirmed by EDX analysis, revealing the presence of Si and Ag in the whole scanned area (S3 Fig). With the aim of having qualitative information about the thickness of the deposited coating, a treated mini catheter was sectioned using a cryogenic procedure and analyzed using SEM. Fig 11 reports the side views of the treated catheter where the deposited coating is clearly visible. A series of different measurement outlined a coating thickness in the range of 0.5 to 0.8 μm.

**Fig 12:**
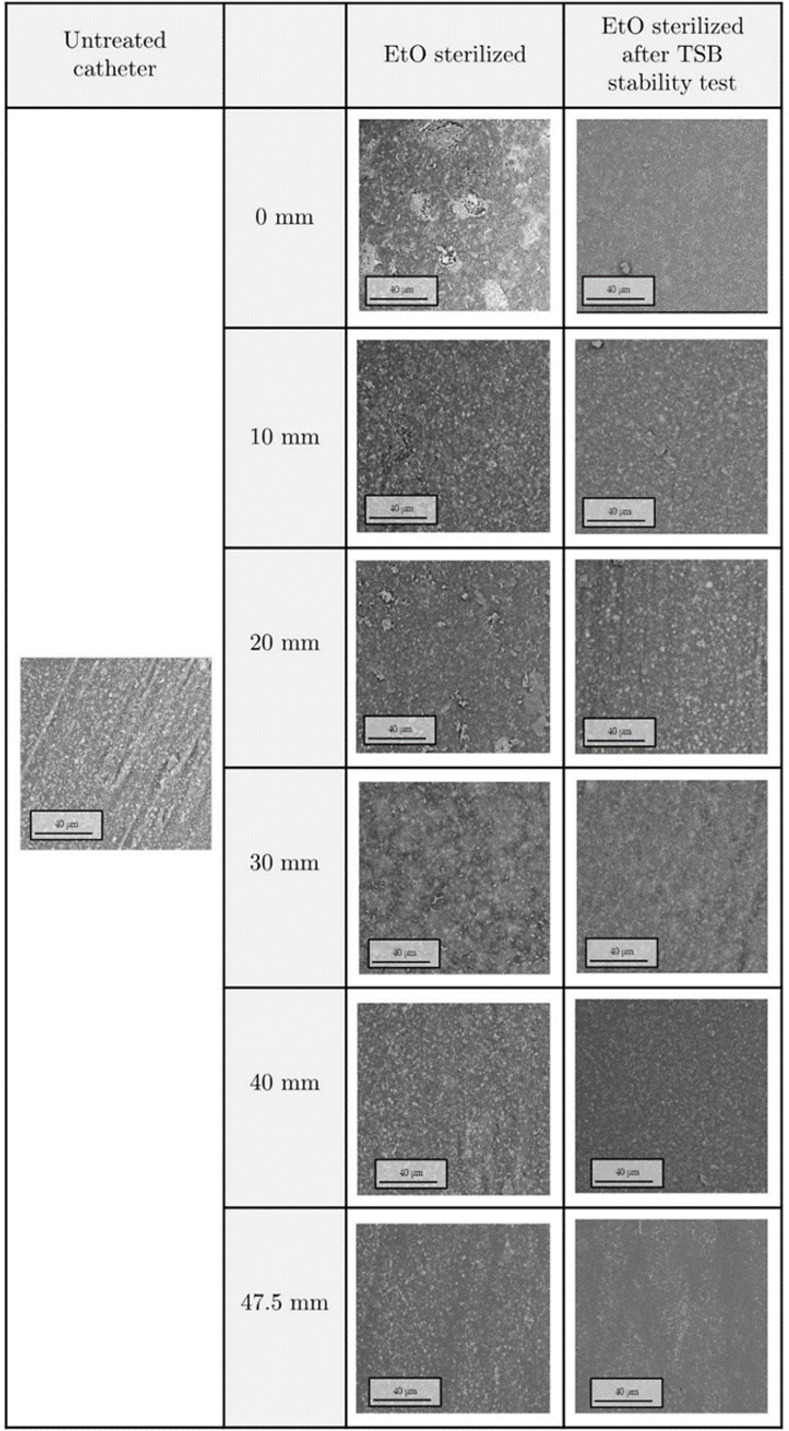
SEM images. Representative SEM images of plasma coated EtO sterilized mini catheter before and after stability test. The length reported in the table is distance from the tip of the catheter.

**Fig 13:**
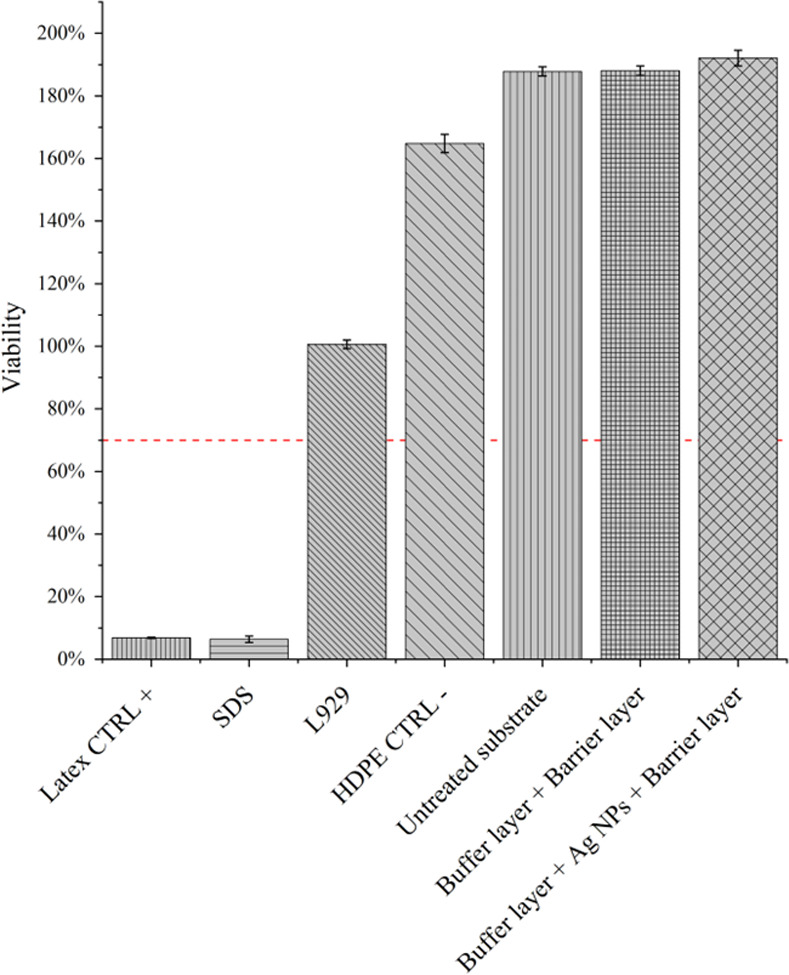
MTT assay. Cell viability from indirect MTT assay as suggested by ISO-10993-5: All the tests were normalized to L922 cell viability. HDPE/Latex and SDS were used as internal and positive and negative cellular control respectively.

EtO treatment was carried out with the aim of removing bacterial contamination from the treated substrates before *in-vivo* application. The stability of the coatings after the sterilization processes was assessed using SEM-EDX techniques, performed before and after sample soaking. Achieved results (Fig 12) highlighted slight modifications of the surface morphology, probably due to a minimal swelling as reported for 2D substrates and a good retain of Ag and Si content (S3 Fig). Once assessed the coating stability upon sterilization and liquid immersion, the biocompatibility of treated catheters was studied. In this perspective, according to regulations, we carried out an MTT test with the aim of evaluating the release of cytotoxic eluates in the liquid environment. It is important to remember that ISO guidelines suggest a minimum viability threshold of 70% to identify non-cytotoxic biomaterials (red dot line). According to our findings reported in Fig 13 and normalized with respect to untreated cell sample, coated substrates displayed a sufficient level of viability with respect to positive control (Latex). The presence of Ag NPs in the nanostructured coatings did not induce significative decrease of cell viability, confirming the good level of hemocompatibility already displayed for the 2D substrates.

**Fig 14:**
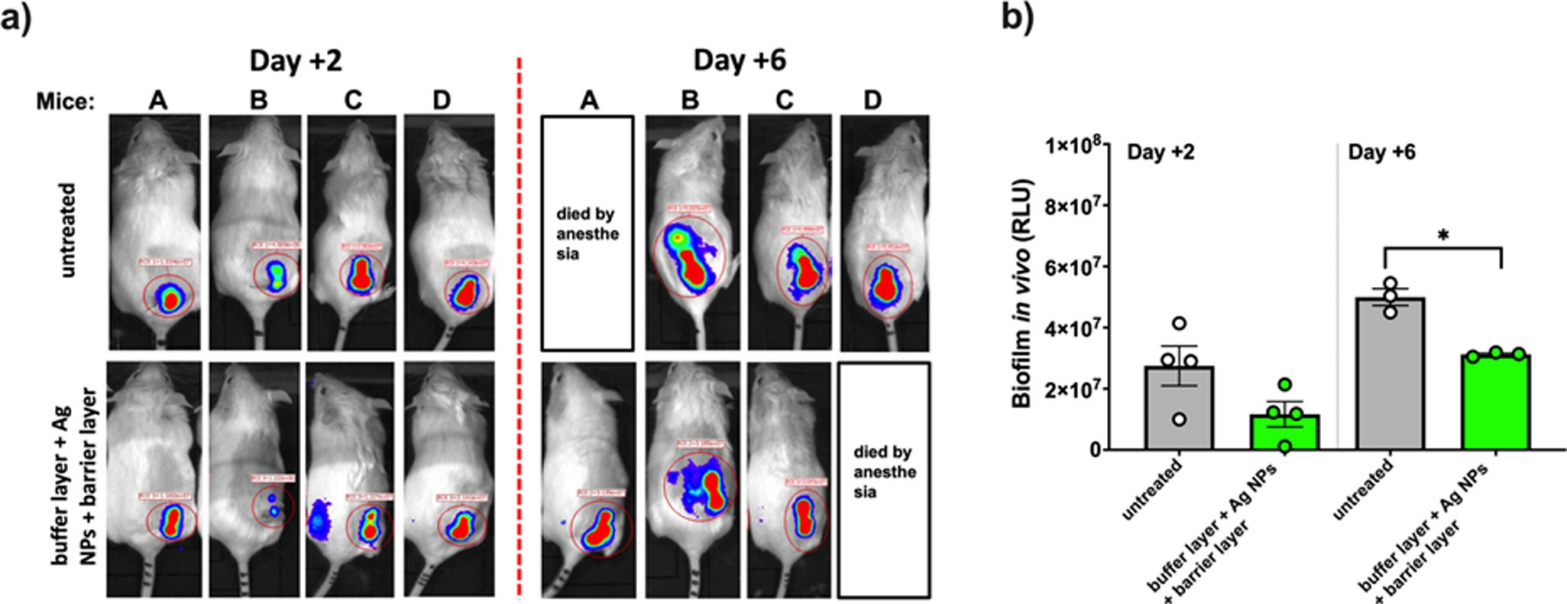
IVIS LUMINA luminescence images. Images of RLU emitted by the Region of Interest of each mouse implanted with infected catheters at day +2 and +6 post implanted (a). The graph shows the mean ± SEM of RLU from the region of interest from each group (b). *p<0.05; buffer layer + Ag NPs + barrier layer catheters vs untreated catheters.

The anti-biofilm properties of untreated and nanostructured coated (buffer layer + Ag NPs + barrier layer) mini catheters were investigated in a murine model of catheter-associated infection by exploiting the bioluminescence emission of an engineered *Pseudomonas aeruginosa* bacterial strain. Once sterilized, mini catheters were contaminated with BLI-*Pseudomonas* as specified in Materials and Methods section and introduced through subcutaneous surgery under mouse skin. Two- and six-days post-implant, mice were anesthetized and imaged using IVIS camera system (Xenogen). Total photons flux emission from the Region Of Interest (ROI) was quantified using Living Image software package (Xenogen) and expressed as Relative Luminescence Units (RLU). Relative Luminescence Units were used to evaluate the amount of biofilm formed on the mouse back, also offering the possibility to real-time monitoring biofilm formation over time in each single animal. The images of each mouse implanted with infected catheters and the graph represent the mean ± SEM of RLU from the region of interest are reported in Fig 14A and 14B, respectively. Even without reaching statistical significance (p = 0.086), the comparison of RLU after 2 days showed that Ag NPs coatings were able to reduce biofilm formation with respect to the untreated catheters. Likewise, the analysis carried out at the sixth day confirmed the consistent anti-biofilm performances of nanostructured coatings, able to significatively limit the infection progression (*p<0.05) (Fig 14B).

## Conclusions

In this study we show the biocompatibility and the antibiofilm properties of nanostructured silver coatings deposited though the atmospheric pressure plasma technology. Ag NPs were synthesized in the plasma discharge and deposited in between two polymeric layers. The organic character of deposited films was confirmed by means of FTIR spectroscopy and CA analysis, while the coating stability upon liquid immersion has been checked through electron microscopy. The presence of Ag NPs has shown to counteract biofilm formation and adhesion on biomaterial surface. Clot formation and hemolysis was also reduced as compared to the untreated biomaterial. This innovative approach (i.e. atmospheric non-thermal plasma assisted deposition of silver-based nanostructured coatings), suitable for in-situ and localized surface functionalization, allowed us to investigate the deposition of multilayer coating on mini catheters. Besides chemical and morphological characterization and EtO stability of treated samples, mini catheters biocompatibility was performed by *in vitro* cytotoxicity and hemocompatibility assays. The absence of any cytotoxic effect granted the possibility to carry on *in-vivo* tests on animal model, showing a positive effect of nanostructured coating deposition in terms of counteracting biofilm formation. In the perspective of future applications, material biocompatibility and coating stability are mandatory requirements that need to be investigated. Although plasma technology offers unique features in terms of material processing and properties tunability, forthcoming applications will gain advantages of a better understanding of the role of coating surface free energy and morphology both on antibacterial properties and on cell compatibility.

## Supporting information

S1 Fig Staining control for haematoxylin/eosin and crystal violet staining. S1 Fig reports the coloration control for dynamic blood and bacterial broth contact tests. Samples were stained according the procedure described in the Materials and Methods paragraph and afterwards stereo microscopy was used to analysed the surface of biomaterials.
(TIF)

S2 Fig EDX spectra of untreated, buffer layer + barrier layer, buffer layer + Ag NPs + barrier layer coatings. S2 Fig reports the EDX spectra of deposited polymeric (buffer layer + barrier layer) and nanostructured coating (buffer layer + Ag NPs + barrier layer) with respect to the untreated sample. While confirming the presence of BaSO4 particles, the characteristic peak of Si was found in both polymeric and nanostructured coating. The latter one also outlined the presence of Ag peak due to NPs embedded in the multilayer structure.
(TIF)

S3 Fig EDX spectra of untreated, plasma treated EtO sterilized mini catheter before and after stability test (all spectra were collected from a region located at 30 mm from the tip of the catheter). EDX spectra collected from mini catheters after EtO sterilization are reported in S3 Fig. Untreated biomaterial outlined the presence of C and O characteristics peaks, confirming the polymeric nature of the substrate. While the presence of Au can be ascribed to the coating procedure (see Materials and Methods paragraph), Ba and S peaks confirmed that during the moulding process the mini catheters were loaded with BaSO4, with the aim of conferring radiopaque properties. When the nanostructured coating was deposited on biomaterials, EDX spectrum outlined the presence of Si and Ag. Finally, the retain of Si and Ag after stability test corroborated materials suitability for in-vivo testing.
(TIF)

S1 Graphical abstract Nanostructured polymeric thin films are deposited with an atmospheric non-thermal plasma jet on flat substrates and on 3D mini catheters. Silver nanoparticles, known for their anti-clot and anti-biofilm properties, are embedded in the coating after being synthesized directly in the plasma jet, converting a silver salt in metallic silver. Biocompatibility, anti-biofilm and anti-clot properties of the coatings are assessed in *in-vitro* and *in-vivo* settings.
(DOCX)
