# Supplementary figures and images for: A new strategy to prevent biofilm and clot formation in medical devices: The use of atmospheric non-thermal plasma assisted deposition of silver-based nanostructured coatings
Source: PLoS One. 2023 Feb 22;18(2):e0282059. doi: 10.1371/journal.pone.0282059 (PMC9946233; doi:10.1371/journal.pone.0282059)

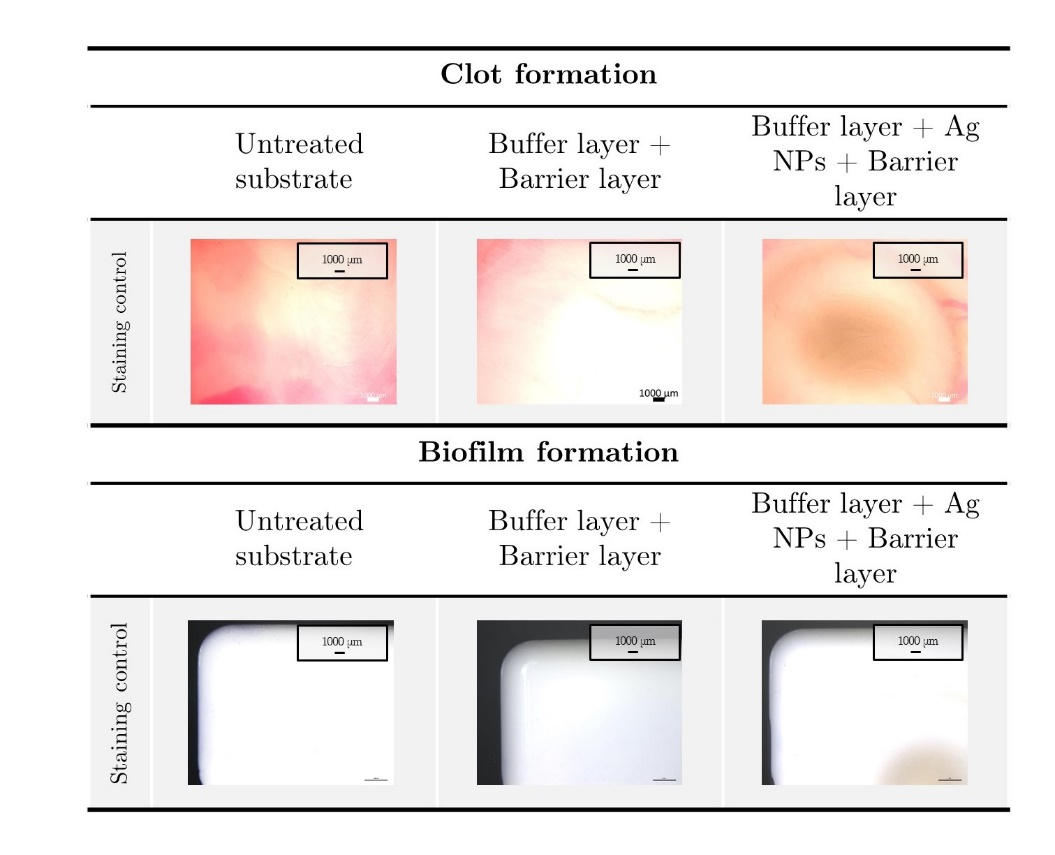

Supplement: S1 Fig — reports the coloration control for dynamic blood and bacterial broth contact tests. Samples were stained according the procedure described in the Materials and Methods paragraph and afterwards stereo microscopy was used to analysed the surface of biomaterials. (TIF) [file pone.0282059.s001.tif]

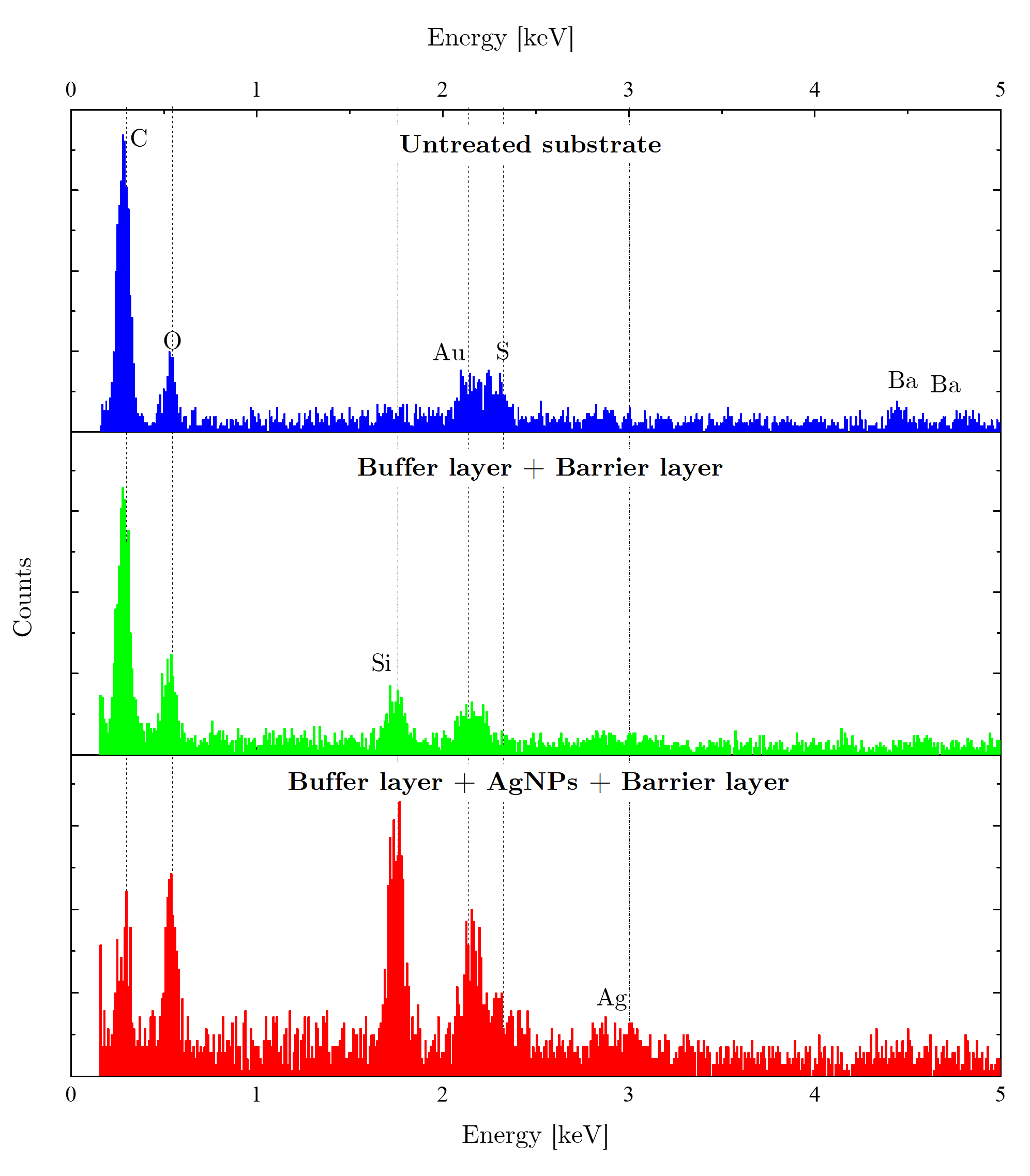

Supplement: S2 Fig — reports the EDX spectra of deposited polymeric (buffer layer + barrier layer) and nanostructured coating (buffer layer + Ag NPs + barrier layer) with respect to the untreated sample. While confirming the presence of BaSO4 particles, the characteristic peak of Si was found in both polymeric and nanostructured coating. The latter one also outlined the presence of Ag peak due to NPs embedded in the multilayer structure. (TIF) [file pone.0282059.s002.tif]

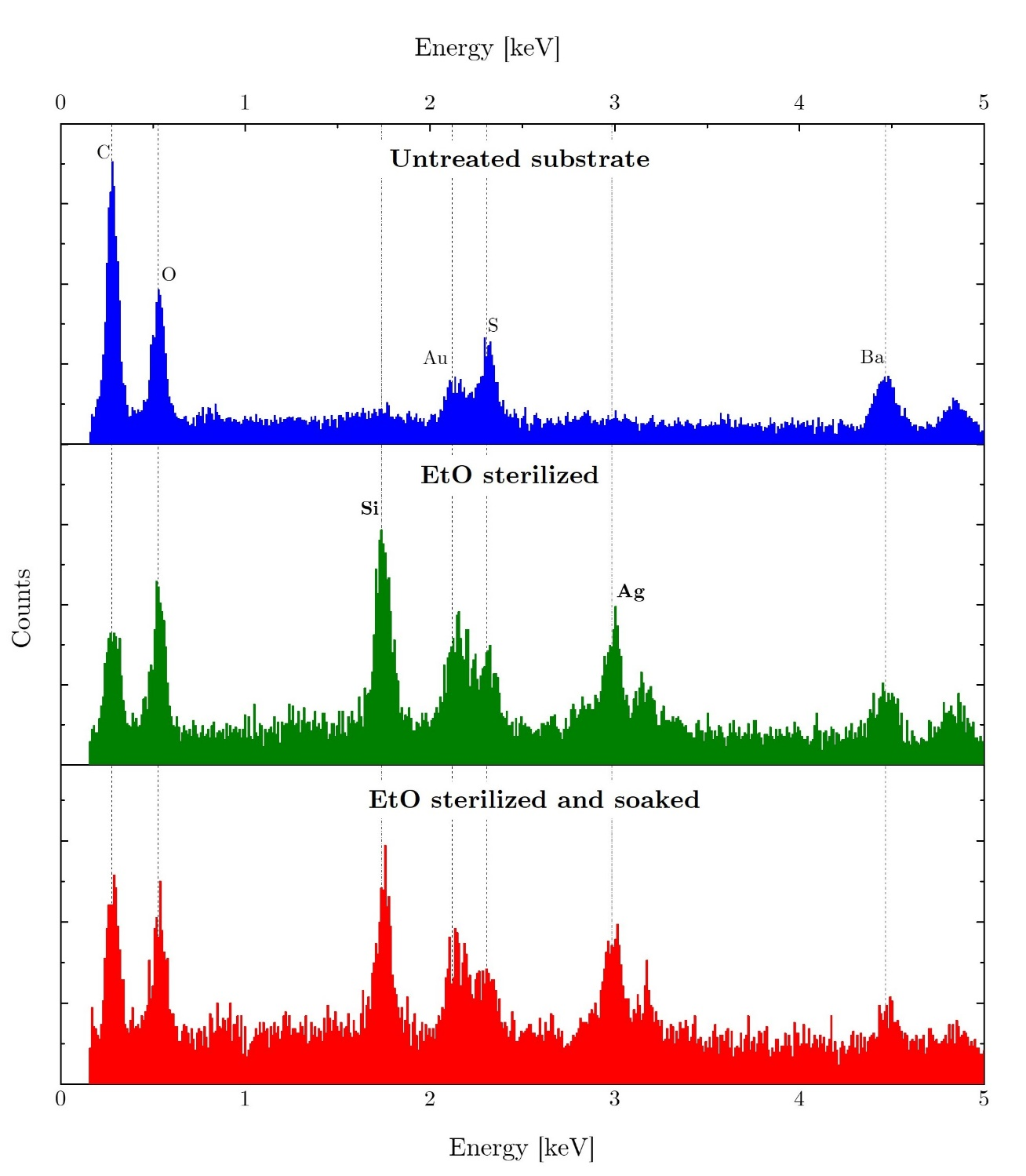

Supplement: S3 Fig — EDX spectra collected from mini catheters after EtO sterilization are reported in S3 Fig. Untreated biomaterial outlined the presence of C and O characteristics peaks, confirming the polymeric nature of the substrate. While the presence of Au can be ascribed to the coating procedure (see Materials and Methods paragraph), Ba and S peaks confirmed that during the moulding process the mini catheters were loaded with BaSO4, with the aim of conferring radiopaque properties. When the nanostructured coating was deposited on biomaterials, EDX spectrum outlined the presence of Si and Ag. Finally, the retain of Si and Ag after stability test corroborated materials suitability for in-vivo testing. (TIF) [file pone.0282059.s003.tif]
