# Supplementary material for: A new strategy to prevent biofilm and clot formation in medical devices: The use of atmospheric non-thermal plasma assisted deposition of silver-based nanostructured coatings
Source: PLoS One. 2023 Feb 22;18(2):e0282059. doi: 10.1371/journal.pone.0282059 (PMC9946233; doi:10.1371/journal.pone.0282059)

**Graphical Abstract**

**Nanostructured polymeric thin films are deposited with an atmospheric non-thermal plasma jet on flat substrates and on 3D mini catheters.** Silver nanoparticles, known for their anti-clot and anti-biofilm properties, are embedded in the coating after being synthesized directly in the plasma jet, converting a silver salt in metallic silver. Biocompatibility, anti-biofilm and anti-clot properties of the coatings are assessed in *in-vitro* and *in-vivo* settings.


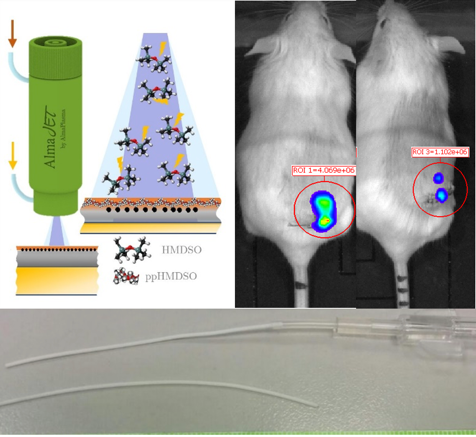

Supplement: S1 Graphical abstract — Silver nanoparticles, known for their anti-clot and anti-biofilm properties, are embedded in the coating after being synthesized directly in the plasma jet, converting a silver salt in metallic silver. Biocompatibility, anti-biofilm and anti-clot properties of the coatings are assessed in in-vitro and in-vivo settings. (DOCX) [file pone.0282059.s004.docx]
